# Validation of World Health Organization Assessment Schedule 2.0 in specialized somatic rehabilitation services in Norway

**DOI:** 10.1007/s11136-016-1384-5

**Published:** 2016-08-09

**Authors:** Vegard Pihl Moen, Jorunn Drageset, Geir Egil Eide, Mari Klokkerud, Sturla Gjesdal

**Affiliations:** 1 Centre for Habilitation and Rehabilitation, Haukeland University Hospital, Østre Nesttunveg 2, 5221 Bergen, Norway; 2 Department of Global Public Health and Primary Care, University of Bergen, Bergen, Norway; 3 Centre for Clinical Research, Haukeland University Hospital, Bergen, Norway; 4 National Advisory Unit on Rehabilitation in Rheumatology, Department of Rheumatology, Diakonhjemmet Hospital, Oslo, Norway

**Keywords:** WHODAS 2.0, Disability, Rehabilitation, Reliability, Validity, Responsiveness

## Abstract

**Purpose:**

The World Health Organization Disability Assessment Schedule (WHODAS) 2.0 is a generic instrument to assess disability covering six domains. The purpose of this study was to investigate the potential of the instrument for monitoring disability in specialized somatic rehabilitation by testing reliability, construct validity and responsiveness of WHODAS 2.0, Norwegian version, among patients with various health conditions.

**Methods:**

For taxonomy, terminology and definitions, the Consensus-based Standards for the Selection of Health Measurement Instruments were followed. Reproducibility was investigated by the intra-class correlation coefficient (ICC) in a randomly selected sample. Internal consistency was assessed by Cronbach’s alpha. Construct validity was evaluated by correlations between WHODAS 2.0 and the Medical Outcomes Study 36-item Short Form, and fit of the hypothesized structure using confirmatory factor analysis (CFA). Responsiveness was evaluated in another randomly selected sample by testing a priori formulated hypotheses.

**Results:**

Nine hundred seventy patients were included in the study. Reproducibility and responsiveness were evaluated in 53 and 104 patients, respectively. The ICC for the WHODAS 2.0 domains ranged from 0.63 to 0.84 and was 0.87 for total score. Cronbach’s alpha for domains ranged from 0.75 to 0.94 and was 0.93 for total score. For construct validity, 6 of 12 expected correlations were confirmed and CFA did not achieve satisfactory fit indices. For responsiveness, 3 of 8 hypotheses were confirmed.

**Conclusion:**

The Norwegian version of WHODAS 2.0 showed moderate to satisfactory reliability and moderate validity in rehabilitation patients. However, the present study indicated possible limitations in terms of responsiveness.

## Background

One of three objectives of the World Health Organization (WHO) Disability Action Plan 2014–2021 is to strengthen the collection of relevant and internationally comparable data on disability [1]. Assessing disability is important for identifying needs when planning healthcare services, setting priorities, allocating resources and evaluating outcomes and effectiveness of interventions [1, 2]. Rehabilitation services target people with various health conditions and disabilities, and optimal functioning is the health goal.

The International Classification of Functioning (ICF), published in 2001, defines functioning and disability in a comprehensive perspective in terms of impairments, activity limitations and participation restrictions, in addition, personal and environmental factors [3]. After the release of ICF, WHO has put in an effort to develop a generic Disability Assessment Schedule (WHODAS) with their latest version 2.0 published in 2010.

WHODAS 2.0 and other instruments assessing disability are summarized in ‘Rehabilitation Measures Database’ [4]. While many instruments primarily focus upon function in primary activities like walking, eating, dressing and grooming, the WHODAS 2.0 also captures function in terms of different social participation activities. Reliable instruments assessing participation is advocated in rehabilitation studies [5, 6]. WHODAS 2.0 was cross-culturally developed and is exclusively based on the ICF component ‘Activity and Participation’ capturing self-perceived disability in six functioning domains defining disability as “a decrement in each functioning domain” [2]. The instrument can be used in general population, indicating a wide range of scores.

WHODAS 2.0 has been applied in surveys of different populations and patient groups using a 36-item version of the instrument, both in homogenous [7–13] and in heterogeneous groups of patients [14–18].

Though WHODAS 2.0 has been used in a wide range of health conditions, it has not been evaluated whether it can serve as a survey instrument for monitoring disability among all patients in specialized somatic rehabilitation services, including whether it is capable of assessing outcomes after rehabilitation. Since no generic instrument assessing disability among all rehabilitation patients has been tested in Norway, comparable data on disability are lacking. WHODAS 2.0 has been translated to Norwegian, and though consensus-based standard guidelines for translation have been followed [19], measurement properties have not been investigated for any health condition. Finally, the original hypothesized structure of the instrument has shown conflicting results in previous studies [14–16, 18].

The aim of the present study was therefore to examine the measurement properties of the Norwegian version of the 36-item version of WHODAS 2.0, as it provides most details, among a heterogeneous sample of patients accepted for specialized somatic rehabilitation. In addition to reliability and validity, responsiveness, which has been less investigated previously, was tested.

## Methods

### Design, setting and patients

The study was based on data from a cross-sectional study of patients from western Norway accepted for specialized somatic rehabilitation between January and June 2015. Patients were invited to participate either by mail from a waiting list or at admission to one of the following institutions: Åstveit Health Center, Red Cross Haugland Rehabilitation Centre, Ravneberghaugen Rehabilitation Centre, LHL Clinics Bergen, LHL Clinics Nærland and Rehabilitering Vest Rehabilitation Centre.

Patients were included if they were at least 18 years old and had sufficient knowledge of the Norwegian language. An informed and written consent was obtained from all individual participants included in the study.

First, all patients completed a set of survey instruments including WHODAS 2.0 and the Medical Outcomes Study 36-item Short Form Health Survey version 1 (SF-36).

Second, to explore the reproducibility of the instrument, a randomly selected sample of patients from the waiting list completed WHODAS 2.0 a second time, within 15 days after first time of completion of WHODAS 2.0 and before admission at rehabilitation institution. Self-perceived change in health status between the two tests was assessed on a five-point Likert scale ranging from much worse to much better.

Third, in order to investigate the responsiveness of the instrument, another random sample of patients recruited at admission, completed WHODAS 2.0 a second time, 4–13 weeks after discharge from the rehabilitation institution. A single global question exploring self-perceived change of activities of daily living, including social participation, after rehabilitation compared to before rehabilitation, was assessed on a five-point Likert scale ranging from much worse to much better.

For taxonomy, terminology and definitions, Consensus-based Standards for the Selection of Health Measurement Instruments (COSMIN) were followed [20].

The study was approved by the regional ethical committee in western Norway, 2014-1636.

### Instruments administered

WHODAS 2.0 is a generic patient-reported instrument that measures health and disability [2]. WHODAS 2.0 exists in a 36-item version and 12-item version with multiple versions with different options for administration [21]. In this study, the 36-item self-administered version was used which covers the following 6 domains: *Cognition* (6 items), *Mobility* (5 items), *Self*-*care* (4 items), *Getting along* (5 items), *Life activities* (8 items) and *Participation* (8 items) [22]. *Life activities* can be divided into activities relating to household (4 items) and activities relating to work/study (4 items). All questions relate to difficulties experienced during the previous 28 days (30 days in the original version). The scores assigned to each item are recoded and summed in each domain with a range from 0 (best) to 100 (worst), using complex scoring (SPSS algorithm is available from WHO) [21]. For people working or studying, all 36 items are calculated to a total score; otherwise, 4 items are omitted. An algorithm enables calculation of domain score of *Life activities* and total score regardless of whether the 4 items relating to work/study are answered.

SF-36 version 1 is a generic patient-reported health survey instrument [23]. The SF-36 comprises 36 questions (items) along eight domains of health: mental health (5 items), vitality (4 items), bodily pain (2 items), general health (5 items), social functioning (2 items), physical functioning (10 items), role limitation related to physical problems (4 items) and role limitation related to emotional problems (3 items). An additional item captures changes in general health over the past year. Twenty questions relate to experiences during the previous 28 days. The response scores for each domain are added, followed by a conversion to a score between 0 and 100 with higher scores indicating better health [23]. The measurement properties of the instrument have been tested extensively [24].

### Statistical analysis

Multiple imputations for missing items were applied according to the WHODAS 2.0 manual [22]. If the rate of missing items was >50 % in WHODAS 2.0 domains or in the total score, data were excluded. Number of imputation sets = 5. Missing items in SF-36 were managed according to the SF-36 manual [23].

Feasibility was assessed by exploring missing items of WHODAS 2.0, and a critical rate of 10 % missing items was used [17]. Scores on WHODAS 2.0 and SF-36 were quantified by the per cent of patients scoring, respectively, the lowest possible or highest possible score in the separate domains and in the total score. Floor effect was defined if more than 15 % obtained the lowest possible score (best for WHODAS 2.0; worst for SF-36), ceiling effect if more than 15 % obtained highest possible score (worst for WHODAS 2.0; best for SF-36) [25].

For reproducibility, **i**ntra-class correlation coefficients (ICC), two-way mixed with absolute agreement, were calculated for domain scores and total score for patients reporting no change in health status. An ICC > 0.70 was regarded as acceptable [25]. Smallest detectable change (SDC) for domains and total score was estimated [25].

Internal consistency was estimated by Cronbach’s alpha coefficient. A coefficient between 0.70 and 0.95 is considered satisfactory [25].

Construct validity was explored by testing hypotheses formulated in advance, comparing WHODAS 2.0 domains to SF-36 domains. Expected correlations between all domains of WHODAS 2.0 and SF-36 domains were defined by authors VPM and MK individually, and the overall agreement of the expected correlation was 72.9 % (35 of 48 correlations). Hypotheses about twelve correlations were chosen for the analysis; the intervals for expected correlations were: <0.3, between 0.3 and 0.6, and >0.6. If fewer than three (25 %) of the hypotheses were rejected, construct validity of WHODAS 2.0 was considered high, and for moderate validity 25–50 % and for low validity, more than 50 % should be rejected [26]. Pearson’s correlation coefficients were estimated.

In addition to comparing WHODAS 2.0 to SF-36, the structural validity was assessed by testing if data (without items concerning work and study) fitted the original hypothesized structure of WHODAS 2.0 with confirmatory factor analysis (CFA). Cut-off close to 0.95 or higher for comparative fit index (CFI), cut-off close to <0.06 or lower for root-mean-square error of approximation (RMSEA) and cut-off close to 0.08 or lower were used to define a satisfactory fit of model [27].

Responsiveness was explored by testing eight hypotheses formulated in advance with the same satisfactory cut-off as construct validity. Three hypotheses included groups of patients in which a change was assumed, expecting the instrument would capture this change when compared to a group of patients where no or small change would occur. Two hypotheses addressed the individual level in a group of patients, one comparing the total score after rehabilitation to before; the second referred to the domain that was assumed to have the greatest change. Furthermore, three hypotheses were based on expected correlations with SF-36 when assessing construct validity. Two of the eight hypotheses addressed those patients who had undergone surgical treatment during the last 4 weeks before admission to the rehabilitation institution, since these patients were expected to have an improvement regardless of rehabilitation.

To complement the method assessing responsiveness using a priori formulated hypotheses, overall change score, effect size (ES) and standardized response mean (SRM) were calculated for domains and total score of WHODAS 2.0 and SF-36 [28–30]. An ES of 0.2 is regarded as low, 0.5 as moderate and 0.8 as high [31].

SPSS for Windows version 22.0 (SPSS Inc., Chicago, IL, USA) was used [32] for all statistical analyses except for the confirmative factor analysis where RStudio 099.879 with Lavaan package 05-20 was used. A significance level of 0.05 was chosen in all statistical tests.

## Results

After exclusion of 31 patients due to missing data in WHODAS 2.0, items completed <16, 970 patients were included in the study. Table 1 shows diagnoses according to the International Classification of Diseases version 10 (ICD-10) categories of the patients, age and female percentage for the three samples: total, reproducibility and responsiveness. The largest proportional (61.5 %) was referred to rehabilitation from their general practitioner, 27.7 % from hospital and 4.8 % from other practitioners (missing = 5.9 %). Fourteen per cent had undergone surgical treatment during the last 4 weeks when completing WHODAS 2.0 the first time and 82.0 % reported some kind of pain. Most (98.4 %) of the questionnaires were completed by the patients themselves. For 452 patients, the 36-item version of WHODAS 2.0 was used, as all items in *Life activities* were completed, whereas for 518 patients the 32-item version was used.

**Table 1:** Distribution on age, sex and ICD-10 categories among included patients accepted for specialized somatic rehabilitation

	Total sample (*n* = 970)	Reproducibility sample (*n* = 53)	Responsiveness sample (*n* = 104)
	*n* (%)	*n* (%)	*n* (%)
Age, mean (SD)	57.8 (14.1)	59.2 (13.4)	59.2 (13.5)
Female	613 (63.2)	34 (64.2)	68 (65.4)
Type of health condition			
Diseases of the musculoskeletal system and connective tissue	455 (46.9)	31 (58.5)	37 (35.6)
Diseases of the circulatory system	185 (19.1)	9 (17.0)	23 (22.1)
Diseases of the nervous system	83 (8.6)	5 (9.4)	7 (6.7)
Neoplasms	50 (5.2)	3 (5.7)	8 (7.7)
Endocrine, nutritional and metabolic diseases	37 (3.8)		5 (4.8)
Diseases of the respiratory system	36 (3.7)	3 (5.7)	5 (4.8)
Injury, poisoning and certain other consequences of external causes	26 (2.7)		3 (2.9)
Diseases of the skin and subcutaneous tissue	24 (2.5)		3 (2.9)
Factors influencing health status and contact with health services	23 (2.4)		6 (5.8)
Mental and behavioural disorders	13 (1.3)	2 (3.8)	1 (1.0)
Other	38 (3.9)		6 (5.8)

*ICD-10* International Classification of Diseases version 10, *SD* standard deviation

Table 2 shows descriptive statistics, missing percentage and reliability coefficients of WHODAS 2.0 and SF-36.

**Table 2:** Distribution and reliability of the WHODAS 2.0 and the SF-36 for patients accepted for specialized rehabilitation in western Norway between January and June 2015

Domain	*N*	Mean (SD)	Observed range	Floor (0) score %	Ceiling (100) score %	Missing domain %	Cronbach’s *α*	Test–retest ICC^a^
WHODAS 2.0 [from 0 (best) to 100 (worst)]								
Cognition	950	17.8 (18.8)	0–90.0	27.6	0.0	2.7	0.87	0.81
Mobility	962	33.8 (25.8)	0–100.0	13.8	0.7	1.5	0.85	0.84
Self-care	968	12.0 (17.6)	0–100.0	53.7	0.1	0.6	0.77	0.63
Getting along	966	24.8 (20.9)	0–100.0	17.2	0.1	2.7	0.75	0.79
Life activities	963	45.1 (27.3)	0–100.0	7.1	4.0	27.4	0.91	0.78
Life activities: household	963	44.8 (27.5)	0–100.0	10.0	4.9	0.8	0.87	0.78
Life activities: work/study	452	42.9 (31.8)	0–100.0	13.8	12.1	1.7	0.94	0.71^b^
Participation	952	41.1 (20.6)	0–100.0	2.1	0.1	3.1	0.83	0.75
Total score	970	30.9 (16.2)	0– 90.2	0.5	0.0	7.9	0.92	0.87
SF-36 [from 0 (worst) to 100 (best)]								
Mental health	957	66.4 (19.3)	0–100.0	0.2	1.4	2.0	0.85	
Vitality	965	33.4 (20.3)	0–100.0	5.8	0.3	2.1	0.82	
Bodily pain	965	40.4 (25.8)	0–100.0	6.7	5.9	1.0	0.88	
General health	945	48.6 (22.2)	0–100.0	0.7	0.4	2.8	0.76	
Social functioning	969	55.4 (28.0)	0–100.0	5.1	10.4	2.0	0.85	
Physical functioning	959	53.3 (25.5)	0–100.0	1.8	1.8	1.6	0.90	
Role physical	940	16.6 (29.4)	0–100.0	67.8	6.7	3.8	0.80	
Role emotional	930	44.1 (43.2)	0–100.0	41.1	31.9	4.5	0.85	

*WHODAS* World Health Organization Disability Assessment Schedule, *SF-36* Medical Outcomes Study 36-item Short Form Health Survey, *SD* standard deviation, *ICC* intra-class correlation

^a^For test–retest, there were 53 patients analysed in the study

^b^For Life activities: work/study, there were 21 patients analysed for ICC

The number of missing was below the critical rate (10 %) in all items of WHODAS 2.0, 0.3–5.5 %, except the item concerning sexual activity (10.4 %) and items concerning *Life activities* *work/study* (53.3–55.2 %). Considering that four items are excluded for those who do not work or study, missing items of the total score were 2.2 % and for *Life activities* *work/study* 0.2–3.8 %.

Ceiling effect was not present in any domains. Floor effect was present in *Cognition*, *Self*-*care* and *Getting along*, with the highest percentage in *Self*-*care* (53.7 %).

Fifty-three patients completed WHODAS 2.0 a second time reporting no change in health status between tests; test–retest period was 7–15 days with mean of 11.6. Missing were lower for retest compared to test, between 0 and 3.0 % for domains.

SDC for the different domains ranged from 22.8 to 35.8 and was 16.2 for the total score.

Table 3 presents the correlation between WHODAS 2.0 domains and SF-36 domains, including the expected correlations marked in ‘bold’. The correlations were negative due to opposing best scores. Six out of twelve hypotheses were confirmed.

**Table 3:** Construct validity as measured by Pearson’s correlation^a^ (*r*) for WHODAS 2.0 versus SF-36 for 970 patients accepted for specialized rehabilitation in western Norway between January and June 2015^b^

	WHODAS 2.0					
	Cognition	Mobility	Self-care	Getting along	Life activities	Participation
SF-36						
Mental health	−0.475	−0.188	−**0.184L**	−**0.476M**	−0.324	−0.547
Vitality	−0.392	−0.312	−0.175	−0.365	−0.440	−0.495
Pain	−0.170	−0.507	−0.293	−0.156	−**0.380M**	−**0.436L**
Physical functioning	−**0.146L**	−**0.764H**	−0.498	−0.110	−0.488	−0.432
Role physical	−0.140	−**0.298M**	−0.167	−**0.159L**	0.417	−0.367
Role emotional	−0.296	−0.153	−0.145	−**0.265M**	−0.254	−0.380
Social functioning	−0.419	−0.440	−**0.328L**	−0.451	−0.542	−**0.660M**
General health	−**0.319L**	−0.325	−0.222	−0.326	−0.333	−0.471

*WHODAS* World Health Organization Disability Assessment Schedule, *SF-36* Medical Outcomes Study 36-item Short Form Health Survey; *L* = *r* < 0.3 expected; *M* = 0.3 < *r* < 0.6 expected; *H* = *r* > 0.6 expected

^a^All correlations had *p* < 0.001

^b^A priori formulated hypotheses marked in bold

**Fig. 1:**
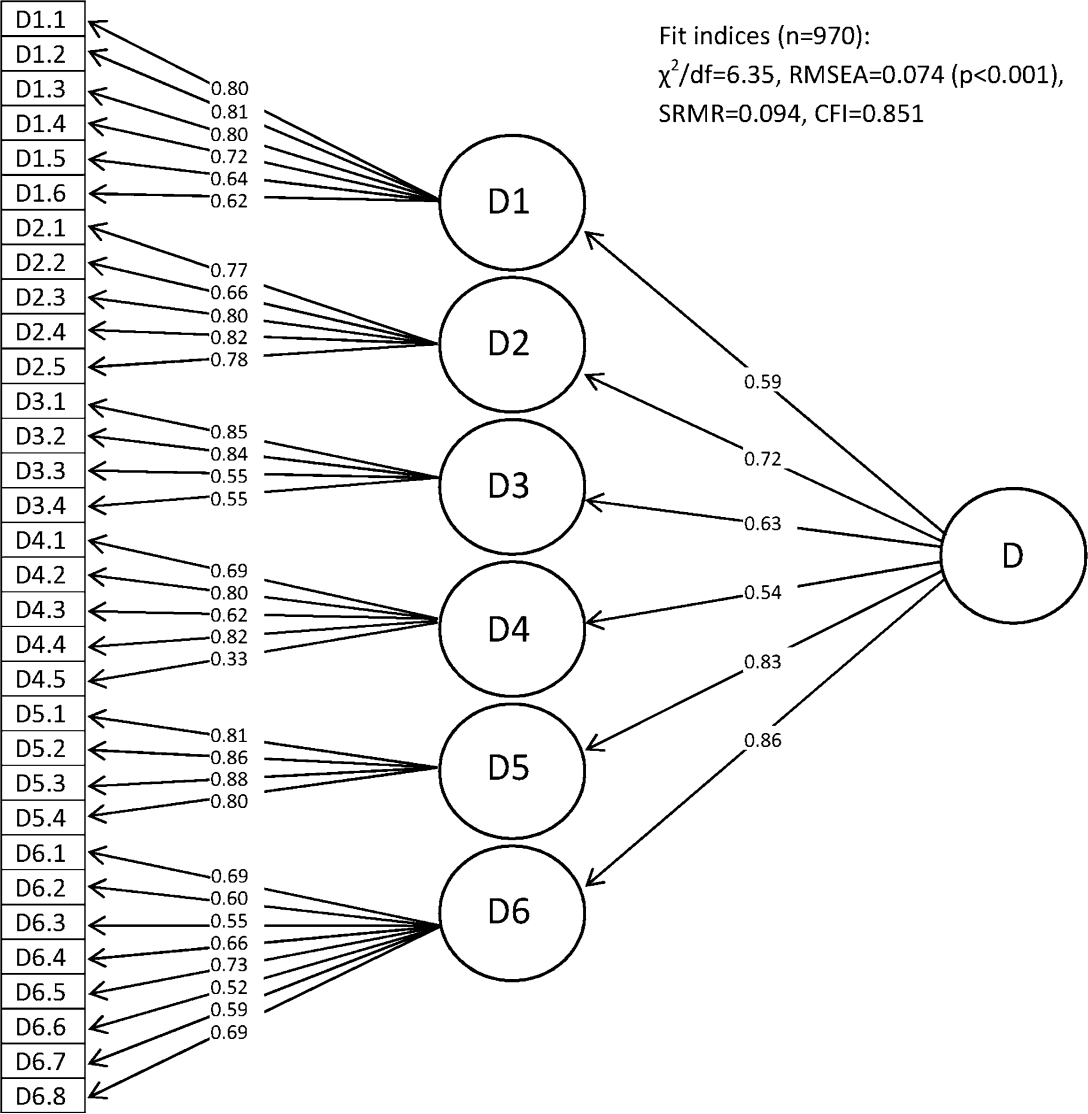
Standardized parameter estimates from confirmatory factor analysis: second-order 6-factor model. D1, cognition; D2, mobility; D3, self-care; D4, getting along; D5, life activities; D6, participation; D, total score/disability

For the 32-item version of WHODAS 2.0, excluding items concerning work and study, the standardized parameter estimates and fit indices for the second-order 6-factor model are shown in Fig. 1.

**Fig. 2:**
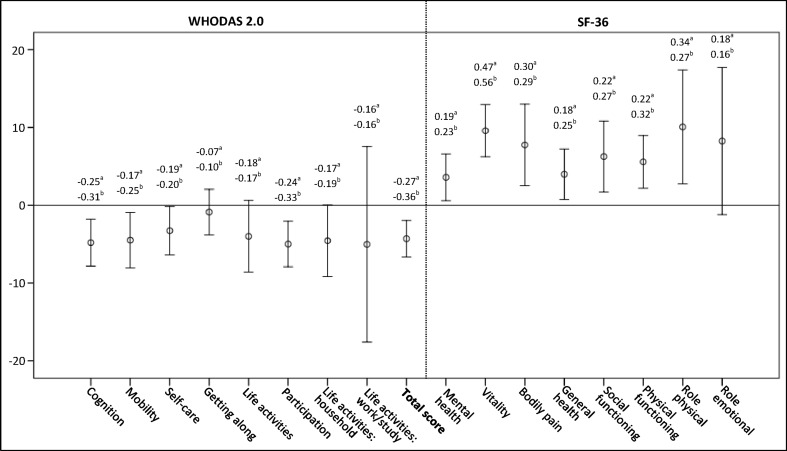
Mean and 95 % CI of overall change score of WHODAS 2.0 and SF-36, domains and total, for rehabilitation patients 4–13 weeks after discharge from a rehabilitation institution compared to admission to the institution (*n* = 104). Change scores are opposite due to opposite best scores. ^a^Effect size, ^b^standardized response mean

One hundred four patients completed WHODAS 2.0 a second time. Mean duration between these assessments were 48.4 days, ranging from 4 to 13 weeks after discharge from the rehabilitation institution. Missing was lower compared to the first time of completion, between 0 and 3.5 % for domains. The result from the single global question (1 missing: *n* = 103) was as follows: 10.7 % reported worse (combining ‘Worse’ and ‘Slightly worse’), 35 % no change and 54.4 % better (combining ‘Slightly better’ and ‘Better’). A percentage of 19.4 reported surgical treatment during the last 4 weeks before admission to the rehabilitation institution. Change score, ES and SRM for WHODAS 2.0 and SF-36 are presented in Fig. 2. All change scores were <SDC for their, respectively, domains or total score. Nonparametric tests were performed since the data, i.e. domain scores and total score before and after rehabilitation, were not normally distributed. Table 4 presents the hypotheses with the results; five of eight hypotheses were rejected.

**Table 4:** A priori hypotheses for examining the responsiveness of the WHODAS 2.0 for 104 rehabilitation patients, statistical results and if confirmed

	Hypotheses	Results	Confirmed
1.	Patients reporting positive change in global question have higher negative change scores in WHODAS 2.0 total score compared to patients reporting no change	*Z* = −0.99; *p* = 0.349^a^	No
2.	Patients reporting negative change in global question have higher positive change scores in WHODAS 2.0 total score compared to patients reporting no change	*Z* = −0.67; *p* = 0.506^a^	No
3.	Patients reporting positive change in global question have lower WHODAS 2.0 total score after rehabilitation compared to WHODAS 2.0 total score before rehabilitation	*Z* = −3.13; *p* = 0.002^a^	Yes
4.	Patients reporting positive change in global question have lowest *Z* value and lowest *p* value in *Mobility* compared to other domains of WHODAS 2.0 after rehabilitation	Cognition: *Z* = −3.05; *p* = 0.002^a^ Mobility: *Z* = −2.11; *p* = 0.035^a^	No
5.	Patients reported to have undergone surgical treatment during the last 4 weeks have higher negative change scores in WHODAS 2.0 *Mobility* compared to patients reported no operation	*Z* = −2.08; *p* = 0.038^a^	Yes
6.	Patients reported to have undergone surgical treatment during the last 4 weeks: change in WHODAS *Mobility* correlates with change in SF-36 physical functioning, correlation lower than −0.5	*R* = −0.169; *p* = 0.496	No
7.	The correlation of change on WHODAS 2.0 *Mobility* on SF-36 physical functioning is at least 0.3 lower than the correlation of change on WHODAS 2.0 participation with SF-36 pain	*R*: −0.194 versus −0.394*	No
8.	The correlation of change on WHODAS 2.0 participation on SF-36 social functioning is at least 0.1 lower than the correlation of change on WHODAS 2.0 cognition on SF-36 physical functioning	*R*: −0.470* versus −0.125	Yes

*WHODAS* World Health Organization Disability Assessment Schedule, *SF-36* Medical Outcomes Study 36-item Short Form Health Survey, *R* Pearson’s correlation coefficient

^a^Two-tailed asymptotic *p* value from Mann–Whitney’s *U* test

* *p* < 0.01

## Discussion

Numerous instruments can be used to assess disability and other health concepts in patients. However, WHODAS 2.0 captures functioning in activities and social participation using the ICF, which is internationally acknowledged, as the conceptual framework. In this study, the measurement properties of WHODAS 2.0, Norwegian version, have been tested to evaluate its potential as an instrument monitoring disability in somatic rehabilitation setting.

Most important, the study supported the results from previous studies of WHODAS 2.0 found in different language versions and populations with moderate to satisfactory reliability, moderate validity and low responsiveness. Our results support the use of WHODAS 2.0 in rehabilitation; however, some considerations should be taken when evaluating outcomes with the instrument.

The Cronbach’s alpha was all above 0.7 indicating satisfactory internal consistency which is consistent with other studies including similar groups of patients [9–12, 17, 18, 33, 34]. The ICC of the different domains and for the total score indicated acceptable reproducibility except for *Self*-*care*. Since ICC is strongly influenced by the variance, low variability in this domain is indicated. In other studies, the reproducibility has been reported with ICC between 0.62 and 0.97 [7, 9, 12, 16, 18]. Reaching the satisfactory cut-off of 0.7, for both Cronbach’s alpha and ICC, supports the use of WHODAS 2.0 for group comparison. However, for individual comparison, including use in clinical practice, an ICC as high as 0.9 is required [35].

The overall low level of missing items indicated high feasibility of WHODAS 2.0. The missing rate above the critical rate of 10 % in the item concerning sexual activity has also been reported in other studies [17, 18]. The possible causes may be that the item is irrelevant for some, or that sexual activity is considered a private issue. The high missing rate in items concerning *Life activities work/study* is due to the fact that many patients had not been working or studying the last 4 weeks prior to completing WHODAS 2.0.

While ceiling effect in *Life activities* *work/study* has been reported previously in patients with chronic diseases [16], no such effect was found in this study, although *Life activities work/study* had the highest proportion of ceiling scores at 12.1, approaching the threshold of 15 %. Floor effects, which have been reported in previous studies [9, 14, 16, 18], were present in three of six domains, implying problems with respect to differentiating patients with low grades of disability. The high floor effect in *Self*-*care* indicates a high degree of self-reliance in the study population as expected as this is an admission criterion for the largest proportion of patients to these institutions. The low percentage of ceiling and floor scores seen in total score and the domain *Participation*, and to some degree *Life activities,* supports the use of these scores in rehabilitation studies in heterogeneous patient populations.

Based on Pearson’s correlations, the number of supported pre-defined hypotheses, the construct validity was considered to be moderate compared to SF-36. Moderate and strong correlations, both expected and not predefined, between the domains of WHODAS 2.0 and SF-36 have been reported previously [7, 8, 15–17]. A method which has been utilized in two studies [15, 17] is grouping the domains of the WHODAS 2.0 and SF-36 into ICF dimensions: “Impairment”, “Activity” and “Participation”. Low, moderate and high correlations between the domains of these instruments grouped into “Activity” or “Participation” have been reported in these studies. A supplementary analysis was conducted adopting this method with their cut-offs to data of the present study. It resulted in 9 low, 2 moderate and 1 high correlations from Table 3, indicating that the domains in these instruments measure different aspects of the ICF dimensions or other health concepts. The use of both instruments when assessing the health status of rehabilitation patients is recommended.

The CFA of a second-order 6-factor model did not reach a satisfactory fit, indicating some degree of misfit. The item concerning sexual activity has also been reported as the lowest parameter estimate in a previous study and the authors suggested a cultural problem [14]. We have no indication that this is a problem in our study sample. In an adjusted model of WHODAS 2.0, with exclusion of the item concerning sexual activity, the fit indices did not differ considerable (data not shown), suggesting retaining this item. The fit indices for a first-order 6-factor model of the 32 items were slightly closer to satisfactory cut-off (data not shown). The findings are somewhat consistent with other studies which have reported fit indices not reaching the proposed satisfactory cut-off used in this study [14, 16, 18], and one study suggested improvement of the structure relocating some items [16]. The lack of consistency with original developers of WHODAS 2.0 may indicate future investigation of the structure, as also a two higher-order factors structure with three domains each has been proposed in patients with depression and low back pain [15]. However, to compare data with other studies using WHODAS 2.0, the original structure should be retained.

The definition and assessment of responsiveness is debated [30]. To our knowledge, this is the first paper evaluating responsiveness of WHODAS 2.0 by testing a priori hypotheses. Results from our study showed low responsiveness related to this study population and its time period, 4–13 weeks.

Though distribution-based methods have some limitations in terms of assessing responsiveness [36, 37], these are often used. The ES reported in the present study was similar and lower compared to previous studies [11, 15–17] which may be explained by shorter assessment period and assessing a heterogeneous group of patients in this study. Low responsiveness was present for *Cognition*, *Participation* and total score if ES is considered to reflect responsiveness. The ES in *Getting along* (−0.07) may indicate a limited impact of rehabilitation on this domain. Since the domains have varying degrees of relevance for different groups of patients, and considering rehabilitation focuses on the individual with individual goals, change scores of the domains between these patients vary as reported in a previous study [17]. However, as ES and SRM are the observed change, results from ES and SRM will not be accurate if responsiveness is considered as the ability of an instrument to detect change [36]. Nevertheless, the results from the present study may indicate low suitability of WHODAS 2.0 for measuring short time changes after discharge from a rehabilitation institution. In addition, the higher SDC compared to change scores found in this study indicates that measuring change with WHODAS 2.0 beyond measurement error might be difficult.

### Strengths and limitations of this study

The large sample size is an important strength, data collected from a prospective study inviting all patients accepted for specialized rehabilitation in western Norway. The sample size of reproducibility was above the number (*n* = 50) recommended as a minimum [25], however, lower than some comparable studies [9, 16].

The heterogeneity of the study population was expected since rehabilitation targets various health conditions. However, one previous study found different correlations of WHODAS II and SF-36 between different health conditions when assessing construct validity [17], entailing difficulties when preparing a priori hypotheses among a heterogeneous population.

Several considerations must be taken into account when interpreting the result of responsiveness. *Mobility* was included in three of eight hypotheses as this domain was expected to improve greatest in most patients during the assessment period. This may have underestimated the responsiveness. Four hypotheses were based on the global question, and this question may be too comprehensive for evaluating change in domains of WHODAS 2.0. Further, the responsiveness was tested with data collected between 4 and 13 weeks after discharge from rehabilitation institutions. This range may be too wide for measuring short time changes and too early after discharge for measuring change in certain domains [17, 38]. Additional follow-up after 6–12 months would probably provide better information about responsiveness. Furthermore, assessing responsiveness in a more homogenous population might simplify the predefined hypotheses. Finally, since responsiveness is an aspect of validity, three of the hypotheses may be considered as an evaluation of discriminate validity between known groups.

The lack of objective data on work and study contributed to a high percentage of ceiling score in the four items concerning work and study. Some patients answered these items by mistake by not reading the instructions in WHODAS 2.0, giving a low score in all these items.

Generalization of the results is only possible considering the study population. Most patients accepted for specialized somatic rehabilitation in Norway are expected to eat and wash themselves, excluding more disabled patients. No information about cognitive function was collected, which may influence data in some patients. However, since patients were self-reliant, this is probably a small problem.

## Conclusion

The Norwegian version of WHODAS 2.0 showed moderate to satisfactory reliability and moderate construct validity compared to SF-36. There is some degree of misfit in the structural model, and there may be some limitations concerning the responsiveness. Overall, for surveying disability in cross-sectional studies and collecting comparable data among patients in specialized somatic rehabilitation, WHODAS 2.0 could be a first choice, as the instrument is based on the ICF, is generic and is easy to administer with high feasibility. Moreover, as rehabilitation puts the patient in focus with individual goals, inclusion of patient-specific instruments might be needed when the effects of rehabilitation are measured. Future studies evaluating short- and long-term responsiveness are needed.
